# Impact of chlorine dioxide disinfection of irrigation water on the epiphytic bacterial community of baby spinach and underlying soil

**DOI:** 10.1371/journal.pone.0199291

**Published:** 2018-07-18

**Authors:** Pilar Truchado, María Isabel Gil, Trevor Suslow, Ana Allende

**Affiliations:** 1 Research Group on Quality, Safety and Bioactivity of Plant Foods, CEBAS-CSIC, Campus Universitario de Espinardo, Murcia, Spain; 2 Department of Plant Science, University of California, One Shields Avenue, Mann Laboratory, Davis, CA, United States of America; Wageningen Universiteit en Researchcentrum, NETHERLANDS

## Abstract

The contamination of pathogenic bacteria through irrigation water is a recognized risk factor for fresh produce. Irrigation water disinfection is an intervention strategy that could be applied to reduce the probability of microbiological contamination of crops. Disinfection treatments should be applied ensuring minimum effective doses, which are efficient in inhibiting the microbial contamination while avoiding formation and accumulation of chemical residues. Among disinfection technologies available for growers, chlorine dioxide (ClO_2_) represents, after sodium hypochlorite, an alternative disinfection treatment, which is commercially applied by growers in the USA and Spain. However, in most of the cases, the suitability of this treatment has been tested against pathogenic bacteria and low attention have been given to the impact of chemical residues on the bacterial community of the vegetable tissue. The aim of this study was to (i) to evaluate the continual application of chlorine dioxide (ClO_2_) as a water disinfection treatment of irrigation water during baby spinach growth in commercial production open fields, and (ii) to determine the subsequent impact of these treatments on the bacterial communities in water, soil, and baby spinach. To gain insight into the changes in the bacterial community elicited by ClO_2_, samples of treated and untreated irrigation water as well as the irrigated soil and baby spinach were analyzed using Miseq® Illumina sequencing platform. Next generation sequencing and multivariate statistical analysis revealed that ClO_2_ treatment of irrigation water did not affect the diversity of the bacterial community of water, soil and crop, but significant differences were observed in the relative abundance of specific bacterial genera. This demonstrates the different susceptibility of the bacteria genera to the ClO_2_ treatment. Based on the obtained results it can be concluded that the phyllosphere bacterial community of baby spinach was more influenced by the soil bacteria community rather than that of irrigation water. In the case of baby spinach, the use of low residual ClO_2_ concentrations (approx. 0.25 mg/L) to treat irrigation water decreased the relative abundance of *Pseudomonaceae* (2.28-fold) and *Enterobacteriaceae* (2.5-fold) when comparing treated versus untreated baby spinach. Members of these two bacterial families are responsible for food spoilage and foodborne illnesses. Therefore, a reduction of these bacterial families might be beneficial for the crop and for food safety. In general it can be concluded that the constant application of ClO_2_ as a disinfection treatment for irrigation water only caused changes in two bacterial families of the baby spinach and soil microbiota, without affecting the major phyla and classes. The significance of these changes in the bacterial community should be further evaluated.

## Introduction

Fruit and vegetables harbor large and diverse types of cultivable and non-cultivable microbes on their surface, which are in constant change during cultivation. Factors affecting changes during cultivation have been associated to crop phenology, crop management, but also due to environmental conditions [1,2]. *Proteobacteria*, *Firmicutes*, *Bacteroidetes* and *Actinobacteria* are the most predominant phyla reported to comprise the bacterial community of the edible part of above-soil harvested vegetables, particularly in leafy greens [3–6]. The composition and the relative abundance of the bacterial taxa vary among plants, mostly due to plant species and phenotypes, but also due to environmental conditions during growing, seasonality, the physiological status of the plant and agricultural practices [7–9]. Previous studies have already evaluated the impact that different agricultural practices have in the bacterial community of different crops. For example, William et al., [8] observed significant differences in the bacterial community of lettuce after irrigation using two different irrigation systems (sprinkler and drip irrigation). On the other hand, the use of irrigation water with different microbial quality did not significantly affect the bacterial community of tomato fruit [10,11]. Agricultural (ag) water has been recognized as one of the major potential vectors of enteric human pathogens during primary production [12–15]. Several strategies have been proposed to reduce the risk of foodborne pathogen contamination including, selection of irrigation water sources, application practices and disinfection technologies such as chemical, physical and combined treatments [16–18]. Among these strategies, chlorine-derived compounds and UV-C water disinfection are widely applied in primary production [18]. Sodium hypochlorite is the most commonly used disinfection agent as it is easy to apply, efficient and cheap [17,18]. However, public health concerns have been raised due to inappropriate hyperchlorination of water and the potential health risks associated to the formation and accumulation of disinfection-by-products in the irrigation water which can be subsequently absorbed by the plant [17, 14]. Thus, the selection of more environmentally friendly technologies to reduce and efficiently control the risk of microbial pathogen contamination in irrigation water has become a priority for growers. Another popular disinfectant agent to treat irrigation water is chlorine dioxide (ClO_2_), which is being commercially applied by leafy greens growers in US and Spain [17, 19]. One of the reasons why ClO_2_ has been suggested as an alternative to sodium hypochlorite is because it does not forms trihalomethanes; however the accumulation of chlorate and chlorite may still be of a concern [19].

The effectiveness of ClO_2_ for irrigation water has been demonstrated for plant and human pathogenic microorganisms [20–22]. However, the impact of long-term application of this water treatment on the bacterial community of different agricultural habitats such as water, soil and crops has not been established. In order to develop recommendations and best practice protocols for growers, an evaluation of the potential impacts needs to be performed in well-controlled studies.

Microbial community profiles from specific ecosystems and econiches can be determined using various techniques. Traditionally, conventional culture-dependent microbiological techniques or 16S rRNA clone libraries and fingerprinting methodologies, including temperature/denaturing gradient gel electrophoresis (TGGE and DGGE), have been used to investigate the microbial community composition [23, 24]. More recently next-generation sequencing (NGS) technologies have provided more comprehensive descriptions of bacterial communities due to the increased number of sequence reads and improved bioinformatics pipelines [25–27]. The recent advances in DNA sequencing can help researchers to understand the interactions between plant and soil microbial communities and the qualitative and quantitative responses to different agricultural practices and variable environmental and seasonal influences.

The goal of the present study was (i) to evaluate the continual application of chlorine dioxide (ClO_2_) as a water disinfection treatment of irrigation water during baby spinach growth in commercial production open fields, and (ii) to determine the subsequent impact of these treatments on the bacterial communities in water, soil, and baby spinach. For this purpose, the comparative bacterial communities were profiled using Illumina high throughput NGS. Results obtained in two commercial fields of baby spinach are presented.

## Material and methods

### Experimental set-up

Baby spinach (*Spinacea Oleracea* L.) was grown in two commercial fields (0.5 and 0.8 ha) located in Pozo de la Higuera (Almería, Spain) and across two consecutive trials (October-December 2015 and February-March 2016). The size of the experimental plots was dictated by the need to conduct comparisons on commercial management units. As contiguous fields were not available, the selected two fields were separated by a distance of 500 m. The edaphoclimatic conditions of the two fields were very similar regarding the soil texture and topography. Nevertheless, in order to minimize uncontrollable influences of the geospatial location and field characteristics, one field was the treated field (irrigated with ClO_2_-treated surface water) in one trial and the other was the untreated one (irrigated with untreated surface water) and the treatment assignment was reversed in the second trial. Crop management of soil preparation, seeding, irrigation, and fertilization were consistent with commercial production practices of baby spinach in this area as described previously [28]. Briefly, surface water stored in a lined water reservoir was used for stand establishment and irrigation. This water source was the only one available in these commercial plots. Water reservoirs are commonly used to guarantee water supply throughout the whole irrigation season in arid and semiarid areas. Therefore, when water is available, the water reservoir is filled and the water used during the whole growing season. The quality of the irrigated water has been previously characterized in López-Gálvez et al. [28] and it was catalogued as good based on its low microbial counts, moderate conductivity and the reduced concentration of organic matter. A stable and highly concentrated aqueous solution of ClO_2_ (≈6000 mg/L), commercially known as AGRI DIS® (Servicios Técnicos de Canarias, Las Palmas de Gran Canaria, Spain) was used to treat the irrigation water. The commercial solution was prepared following the manufacturer instructions. The concentrated ClO_2_ solution (6000 ppm) was diluted with irrigation water in a 1000 L opaque plastic tank. The diluted ClO_2_ solution (approx. 100 ppm) was pumped into the irrigation water system using a programmable Venturi suction unit (INTA Crop Technology S.L., Águilas, Spain). Dosing of ClO_2_ was carried out to fulfill the ClO_2_ demand of the irrigation water and to maintain a constant residual dose of about 0.25 ppm, within an interval of 0.2–0.7, but always below 1 mg/L. Monitorization of the ClO_2_ concentration in the irrigation water was determined using the chronoamperometry analysis Chlordioxense (Palintest, Gateshead, UK) with a limit of detection of 0.02 mg/L. Analyses were performed on a daily basis at the sprinkler head during the irrigation event.

### Sampling

Water samples were taken directly from the solid set sprinkler irrigation system. Soil and baby spinach was sampled at the end of the growing season at the commercial maturity stage of the plants (12–15 cm long measured from the petiole and 6 expanded leaves per plant). Plant samples (100 g) were hand harvested using scissors by excision from the base of petioles and stored in sterile plastic bags which were maintained on ice during transportation to the laboratory (aprox. 45 min.). Scissors were wiped with an ethanol (70%) saturated cloth between use on the different fields (ClO_2_ treated and untreated). Soil and baby spinach samples were taken from five representative positions, homogeneously distributed within each field. Plants were harvested at each designated site from an area of 0.5 and 0.8 ha which corresponded to the ClO_2_ treated and untreated fields, respectively. Soil samples (25 g) were taken at the soil surface (no more than 3 cm deep) located around each sampled plant. Irrigation water samples (2.5 L each) were taken from different sprinkler riser positions located at each growing field. About 400 mL of sodium thiosulfate (5 g/L; Sigma-Aldrich, Darmstadt, Germany) was added to quench oxidizer residuals in samples of irrigation water treated with ClO_2_. All samples were transported (approximately 90 km) under refrigerated conditions in polystyrene boxes to the CEBAS-CSIC laboratory (Murcia, Spain), and stored refrigerated at 4°C until further processing. Processing of the samples was performed within the first 12 hours after sampling.

### DNA extraction

Samples of baby spinach (60 g each) were sonicated in 240 mL of 0.2% sterile buffered peptone water (BPW; Scharlau Chemie, Barcelona, Spain) supplemented with 1% of Tween-80 (Polyethylene glycol sorbitan monooleate; Sigma Aldrich, St Louis, MO, USA). Soil samples (3 g each) were stomached in 150 mL of BPW for 1 min. Sonicated baby spinach and stomached soil were centrifuged at 3000 X *g* for 10 min, the supernatant was decanted, and the pellet obtained was stored at -20°C for DNA extraction. For both sample types, extraction processing used the FastDNA® SPIN Kit for soil and the FastPrep® 24-Instrument (MPBiomedicals, Solon, OH, USA), according to the manufacturer's recommendations. Irrigation water samples (500 mL each) were centrifuged at 3000 X *g* for 20 min. As stated above, the resultant pellets were kept at -20°C until the DNA extraction was performed following the previously described protocol [29]. Briefly, the resulting pellets were lysed by enzymatic treatment with Proteinase K (50 μg/μL). For each sample, DNA was extracted from the lysed pellets using the MasterPure™ Complete DNA and RNA purification kit (Epicenter, Madison, USA) according to the manufacturer's instructions. The quality and concentration of DNA extracts were determined by spectrophotometric measurement at 260/280 nm and 260/230nm using a NanoDrop®ND-1000 UV-Vis spectrophotometer (Thermo Fisher Scientific, Inc., Waltham, MA, USA). Limitations can be also related to the selection of the polymorphic hypervariable regions. In the present study, the V3-V4 regions were selected as they have been defined as the most reliable regions for representing the full-length 16S rRNA sequences in the phylogenetic analysis of most bacterial phyla. However, other regions, such as V6, have been suggested by the literature. In the present study, this region was not included due to technical limitations.

### Illumina sequencing

The V3-V4 hypervariable region of the 16S rRNA gene was amplified using primers S-D-Bact-0341-b-S-17/S-D-Bact-0785-a-A-21 [30] with Illumina overhang adapters on a MiSeq (Illumina, Hayward, CA, USA) instrument. The libraries were generated using two limited-PCR cycles. The first one included an initial denaturation step at 98°C for 30 s; 20 cycles of a denaturation step at 98°C for 30 s, an annealing step at 50°C for 20 s, an extension step at 72°C for 20 s; and a final extension at 72°C for 2 min. Once completed, a PCR clean up step was performed using AMPure XP beads (New England Biolabs, Ipswich, MA, USA) to purify the 16S V3 and V4 amplicon, avoiding free primers and primer dimer species. Illumina sequencing adapters and dual-index barcodes were added to the amplicon. To attach dual-index barcodes (S-D-Bact-0341-b-S-17: ACACTGACGACATGGTTCTACA and S-D-Bact-0785-a-A-21: TACGGTAGCAGAGACTTGGTCT), a second PCR was performed using an Illumina sequencing adapter from Nextera XT Index primers, developed by Illumina (Illumina, Hayward, CA, USA). The second thermal cycling step included an initial denaturation step at 98°C for 30 s; 12 cycles of a denaturation step at 98°C for 30 s, an annealing step at 60°C for 20 s, an extension step at 72°C for 20 s; and a final extension at 72°C for 2 min. The obtained PCR products were cleaned with AMPure XP beads before library quantification was performed. The concentrations and qualities of library preparations were determined using the Quant-iT PicoGreen double stranded DNA assay (Invitrogen, Carlsbad, CA, USA). Sequence data were analyzed using the Quantitative Insights into Microbial Ecology (QIIME) program, version 1.9.1 [31]. The output file was processed for quality filtering by split_libraries_fastq.py. High quality sequences were grouped into Operational Taxonomic Units (OTUs) with a sequence identity threshold of 97%. Taxonomy was assigned by interrogating the high quality sequences with the Greengenes database (13_5). Unclassified OTU sequences were manually annotated against the NCBI database using the BLASTn function. Data were randomly subsampled to the sequence count of the sample with the lowest sequence count using rarify_even_depth implemented in the phyloseq package [32]. An average of 5624 reads per sample were obtained and grouped into 803 phylotypes. Average read lengths from all samples were 240 bp. Sequence depths from samples of irrigation water, soil, and baby spinach were determined by the rarefaction curves and shown in the supplementary data (S1 Fig).

### Statistical analysis

All statistical analyses were performed in R-studio program (3.3.2) and IBM SPSS Statistics 23 (SPSS, Chicago, IL). For each sample, total number of species, Fisher’s diversity, Shannon, Simpson and inverse Simpson indices were calculated to assess the alpha diversity. Pielou’s index was used as indicator of evenness in the community. Correlation among samples was assessed using cluster analysis and the metric multidimensional scaling (MDS) ordination method. Bray-Curtis and Jaccard distances were used to construct dissimilarity matrices of the communities. Beta diversity of the community was determined and Nonmetric multidimensional scaling (NMDS) was employed to visualize the differences among samples using the vegan package in R [33]. Dissimilarity analyses of bacterial community structures in samples from different treatments (ClO_2_ treated and untreated) were calculated using the function Adonis (PERMANOVA) and ANOSIM. Differences in alpha diversity, evenness measures and relative abundances of bacteria genera between treated and untreated samples were compared using Mann Whitney U and Kruskal–Wallis tests.

## Results and discussion

### Bacterial community composition of irrigation water, soil and baby spinach

**Fig 1:**
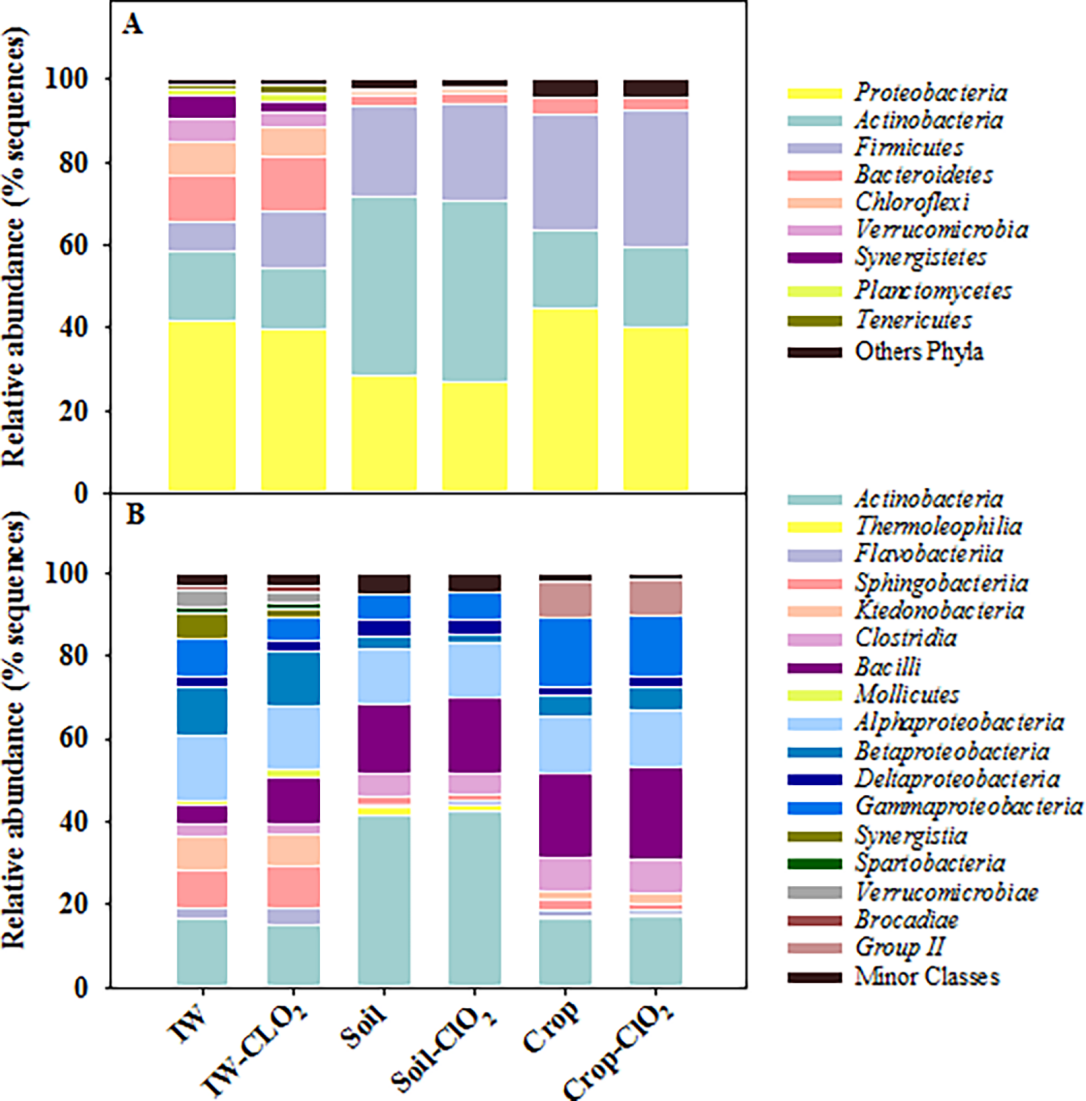
Composition of bacterial phyla and classes of untreated and ClO_2_ treated samples. Percentage of relative abundance of bacterial phyla (A) and classes (B) of untreated and ClO_2_ treated samples of irrigation water (IW), soil (Soil) and baby spinach (Crop). Bacterial community is the average of 5 individual samples. Data shown are phyla that comprised at least 1% of the sequences in at least one sample of a given agronomic habitat.

Based on the protocols utilized, the bacterial community of irrigation water was dominated by *Proteobacteria* (41.53 ± 1.42% average for control samples ± standard deviation) and *Actinobacteria* (16.71 ± 5.41%) followed by less abundant phyla such as *Bacteroidetes* (11.13 ± 3.09%), *Chloroflexi* (7.99 ± 4.33%), *Firmicutes* (7.47 ± 1.33%) and *Verrumicrobia* (5.57 ± 0.78%) (Fig 1A). Regarding classes, *Actinobacteria* (16.52 ± 5.42%), *Alpha* (15.65 ± 6.39%), *Beta* (11.49 ± 1.54%), and *Gammaproteobacteria* (3.51 ± 1.39%) were the most predominant taxa (Fig 1B). The members of the most predominant families in the irrigation water were *Thermogemmatisporaceae* (8.78 ± 3.82%), *Dethiosulfovibrionaceae* (5.20 ± 1.88%), *Pseudonocardiaceae* (4.83 ± 1.06%), *Saprospiraceae* (4.70 ± 1.13%), *Comamonadaceae* (4.61 ± 1.78%), *Verrucomicrobiaceae* (4.15 ± 0.82%) and *Campylobacteraceae* (4.07 ± 0.66%) (S1 Table). These results are consistent with those described for the bacterial community of surface water [34–36].

Among soil samples, the dominant phyla were *Actinobacteria* (43.19 ± 2.90%), followed by *Proteobacteria* (28.28 ± 2.19%), *Firmicutes* (21.84 ± 1.50%*)* and *Bacteroidetes* (2.77± 0.35%) (Fig 1A). These identified phyla, as well as the *Acidobacteria* phyla have been associated previously with the bacterial community of soil in arid and semiarid Mediterranean regions [37–39]. However, the *Acidobacteria* phylum was not detected in the current study. The difference observed in the identified phyla could be due to environmental factors but also due to some intrinsic characteristics of the plant material (e.g. genotype and leaf maturity) [40–42]. Among the identified classes, the predominant ones were *Actinobacteria* (41.31 ± 2.88%), *Bacilli (*16.74 ± 1.72%), *Alphaproteobacteria* (13.45 ± 1.23%), and *Gammaproteobacteria* (6.03 ± 0.49%) (Fig 1B). Further analyses carried out in soil samples revealed the presence of a total of 23 major families (abundance > 1% in at least one sample) and 58 genera (abundance > 0.5% in at least one sample). Among them, the most abundant families represented were *Bacillaceae* (13.49 ± 1.45%), *Nocardioidaceae* (10.38 ± 0.61%), *Micrococcaceae* (9.72 ± 1.35%), and *Streptomycetaceae* (7.21 ± 1.44%), which included members of the genera *Bacillus* (10.30 ± 11.10%), *Nocardioides* (5.92 ± 0.52%), *Arthrobacter* (6.11 ± 0.93%) and *Streptomyces* (6.68 ± 1.37%) (S1 Table).

Schlatter et al. [43] suggested that the bacterial community of plants could be influenced by the soil bacterial community. Supporting this general expectation, Mowlick et al. [44] observed similar bacterial diversity in soil and spinach samples collected from the same production field. Splash dispersal and deposition of soil during irrigation events would be a reasonable mechanism, for a low growing crop like baby spinach, to result in these outcomes. However, generalizations are difficult to make from such a limited sample size, where only one commercial field has been monitored. Additionally, it should be taken into account the complexity of soil microbial community and the myriad of ways in which different climate drivers such as temperature and precipitation might affect soil microorganisms [45].

In baby spinach, the most dominant phyla were *Proteobacteria* (44.64 ± 8.54%), *Firmicutes* (28.29 ± 11.23%), and *Actinobacteria* (18.73 ± 3.95%), accounting for more than 92% of the total sequences (Fig 1A). Additionally, the bacterial classes with the highest relative abundance were *Bacilli* (20.33 ± 6.12%), *Gammaproteobacteria* (16.58 ± 6.0%), *Actinobacteria* (16.55 ± 4.39%), *Alphaproteobacteria*, (13.76 ± 4.21%) and *Betaproteobacteria* (5.08 ± 2.93%) (Fig 1B). These results agree with previous studies that reported these phyla and classes as the predominant ones in the phyllopshere of baby spinach [46,47]. At the family level, *Bacillae* (12.79 ± 3.76%), *Pseudomonaceae* (7.96 ± 4.59%) and *Sphingomonadaceae* (4.51 ± 1.12%) were the predominant families in baby spinach when both irrigated with treated and untreated water (S1 Table). This study is not the first one identifying members of the *Sphingomonadaceae* family in baby spinach phyllosphere. A previous study published by Lopez-Velasco et al., [48] isolated *Sphingomonas*, of the *Sphingomonadaceae* family from spinach leaf surface. However, other families such as *Enterobacteriaceae*, previously described in the phyllosphere of spinach [5,46], were found at low abundance in the present study. This low abundance of the *Enterobacteriaceae* family could be attributed not only to environmental differences but also to some intrinsic characteristics of the plant material such as differences in the genotype and leaf maturity [40–42]. At the genus level, the most prevalent ones in the phyllosphere of baby spinach were *Bacillus* (10.32 ± 3.00%) and *Pseudomonas* (7.96 ± 4.58%). In agreement with other studies on bacterial community of spinach, *Pseudomonas* were the dominant genera detected on surface leaves (4–29% of the population), although *Bacillus* was found at lower concentrations (2.2–0.8% of the population) [5,6]. Additionally, other less prevalent genera (e.g., *Massilia* and *Pantoea*) previously identified as part of the core phyllosphere community of leafy greens [3,4,25,46] were also found in ClO_2_ treated and untreated baby spinach.

### Bacterial community structure of irrigation water, soil and baby spinach due to the ClO_2_ treatment

**Fig 2:**
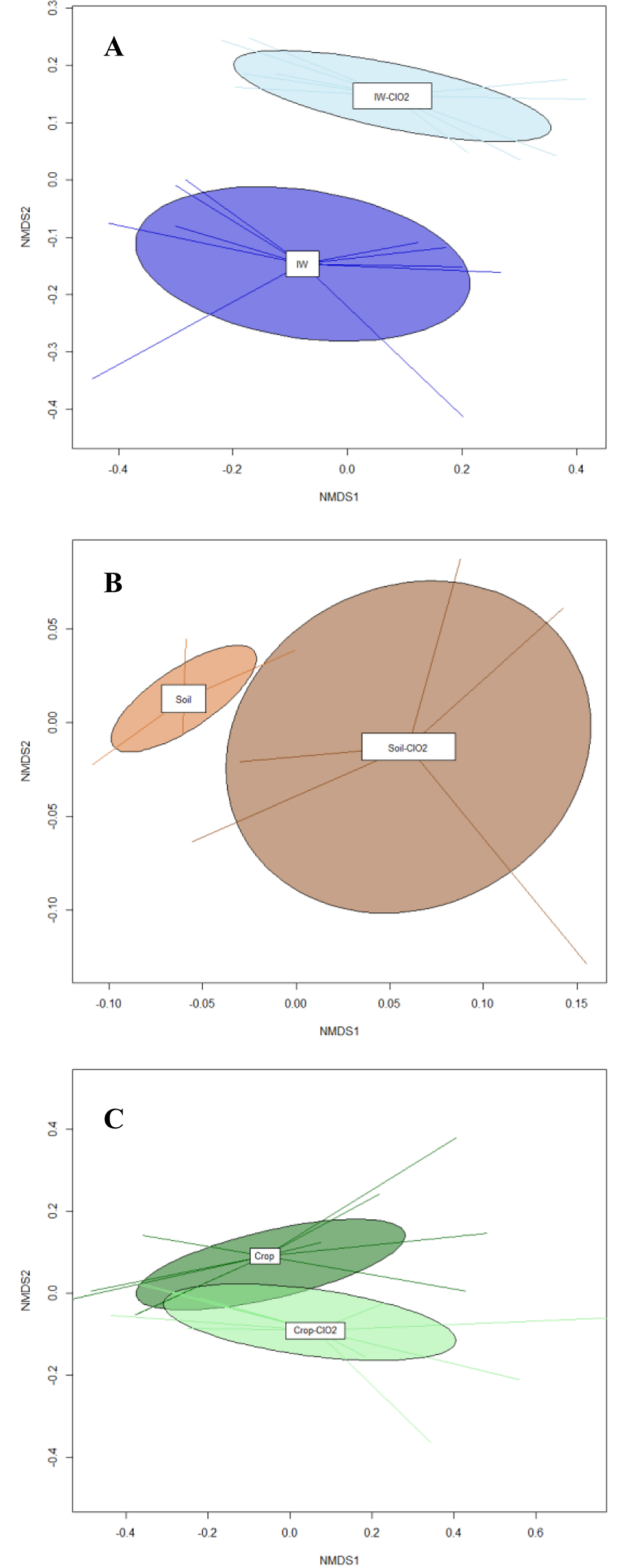
Comparison of bacterial community of untreated and ClO_2_ treated (ClO_2_) samples of irrigation water (IW), soil (Soil) and baby spinach (Crop). Nonmetric multidimensional scaling (NMDS) based on Bray–Curtis distance from OTUs abundance.

In order to determine the impact of ClO_2_ disinfection treatment of irrigation water on the bacterial diversity of different agronomic factors (irrigation water, soil and leaves), the species richness, the alpha-diversity index and evenness were calculated for untreated and ClO_2_ treated samples. The selected indexes did not show significant differences (Mann Whitney test P < 0.01) between treated and untreated samples. These results were based on the similar range of alpha-diversity between samples (**Table 1**). Nonmetric multidimensional scaling (NMDS) plots using Bray-Curtis distance, based on the OTUs matrix, were performed to evaluate the structure of bacterial community from the different agronomic factors. It was observed that NMDS plots for treated and untreated irrigation water and soil samples irrigated with treated and untreated water associated in distinct clusters (Fig 2A and 2B). In contrast, bacterial community of baby spinach irrigated with treated and untreated water clustered together (Fig 2C). The dissimilarity analysis of bacterial community structures of irrigation water, soil and baby spinach revealed that significant differences on the OTUs abundance were only observed between treated and untreated irrigation water samples (Table 2). These results showed that the use of ClO_2_ as a water disinfection treatment had a low impact on the soil and plant bacterial communities.

**Table 1:** Species richness (total species), diversity (Shannon, Fisher’s alpha, Simpson and Inverse Simpson indices), and evenness (Pielou’s index) of untreated and ClO_2_ treated samples of irrigation water (IW), soil (Soil) and baby spinach (Crop).

Index		IW	Soil	Crop
**Total species**	Untreated	523 (514 ± 557)	613 (609 ± 633)	556 (490 ± 607)
	ClO_2_ Treated	558 (534 ± 572)	599 (587 ± 613)	576 (470 ± 6.21)
**Shannon**	Untreated	4.11 (3.97 ± 4.19)	4.67 (4.61 ± 4.78)	3.05 (1.05 ± 4.46)
	ClO_2_ Treated	4.18 (4.05 ± 4.39)	4.73 (4.56 ± 4.78)	2.86 (1.50 ± 0.43)
**Fisher**	Untreated	7.75 (7.50 ± 8.17)	8.70 (8.57 ± 8.76)	8.26 (6.80 ± 9.16)
	ClO_2_ Treated	8.02 (7.77 ± 8.54)	8.85 (8.70 ±9.01)	8.14 (6.74 ± 9.05)
**Simpson**	Untreated	0.95 (0.94 ± 0.90)	0.97 (0.97 ± 0.95)	0.75 (0.42 ± 0.94)
	ClO_2_ Treated	0.96 (0.95 ± 0.97)	0.97 (0.96 ± 0.98)	0.69 (0.42 0.93)
**Inverse Simpson**	Untreated	21.25 (18.03 ± 27.56)	34.01 (33.25 ± 41.32)	6.52 (1.74 ± 19.53)
	ClO_2_ Treated	25.01 (21.27 ± 26.81)	39.48 (32.32 ± 42.47)	13.03 (2.11± 16.15)
**Pielou**	Untreated	0.65 (0.64 ± 0.69)	0.73 (0.72 ± 0.75)	0.48 (0.24 ± 0.70)
	ClO_2_ Treated	0.66 (0.64 ± 0.69)	0.73 (0.71 ± 0.74)	0.45 (0.24 ± 067)

Values are the mean ± SEM of n = 20 for irrigation water, n = 10 for soil and n = 20 for baby spinach.

**Table 2:** Dissimilarity analysis of bacterial community structured in irrigation water (IW), soil (Soil) and baby spinach (Crop).

	Adonis		Anosim	
	*F*	*P*	*R*	*P*
**IW**	5.881	0.01	0.476	0.01
**Soil**	1.866	0.07	0.196	0.08
**Crop**	0.934	0.31	0.008	0.29

### Bacterial community composition of irrigation water, soil and baby spinach due to the ClO_2_ treatment

In the case of irrigation water, no significant differences were found in the relative abundance of the predominant taxonomic groups of bacteria between treated and untreated samples. However, when compared to the untreated water, ClO_2_ treated water showed a reduction of the relative abundance of *Verrucomicrobiae* and *Synergistia* classes of about 1.17 (P<0.000) and 2.53 (P<0.005), respectively (Fig 2). Previous studies in drinking water and wastewater reported that the abundance of the phylum *Proteobacteria* decreased upon chlorine disinfection [49,50]. The differences observed with the present study could be due to the low concentration of ClO_2_ applied for the irrigation water disinfection, which might have affected the relative abundance of less predominant families, without affecting the most predominant ones. A second hypothesis could be associated with the fact that DNA-high throughput sequencing could not distinguish between viable and dead cells. In an attempt to alleviate this limitation, DNA intercalating dyes such as Propidium monoazide (PMA) could have been an efficient methodology to include during sample processing [29,51]. Recently, [52] demonstrated that the use of (NGS) combined with PMA was an efficient technique to determine the changes in the bacterial community composition of drinking water when comparing treated and untreated water disinfection samples.

At genus level, 73 major genera (abundance > 0.5% in at least one sample) were identified in the irrigation water samples, showing 17 of them having significant differences between ClO_2_ treated and untreated irrigation water. Among them, ClO_2_ treatment significantly reduced the relative abundance of phylotypes belonging to genera such as *Acidimicrobium*, *Kribbella*, *Mycobacterium*, *Rhodococcus*, *Saccharopolyspora*, *Euzebya*, *Kaistobacter*, *Novosphingobium*, *Rhodobacter*, *Sphingobium*, *Sphingomonas*, *Polynucleobacter*, *Legionella*, *Luteibacter*, *Dethiosulfovibrio*, *Candidatus* and *Xiphinematobacter* (Table 3). In consequence, the relative abundance of some others increased such as *Demequina*, *Chitinophaga*, *Flavobacterium*, *Fluviicola*, *Bacillus*, *Clostridium*, *Candidatus Scalindua*, *Acidiphilium*, *Polaromonas*, *Campylobacter* and *Mycoplasm* (Table 3). Nevertheless, members belonging to families associated with pathogenic bacteria such as *Legionella* and *Mycobacterium* species were detected, although the abundance of these pathogenic bacteria decreased in irrigation water treated with ClO_2_ (Table 3). These results are in agreement with others that showed the decrease in *Legionella* and *Mycobacterium* when using different disinfection water treatments such as, ozone, chlorine, and ClO_2_ [53–55].

**Table 3:** Bacteria genera that showed significant differences (P < 0.05) in their relative abundances between untreated and ClO_2_ treated samples of irrigation water (IW), soil (Soil) and baby spinach (Crop).

	Taxonomy*								
	Phylum	Class	Order	Family	Genera	*P*	Untreated		ClO_2_Treated
**IW**	*Actinobacteria*	*Acidimicrobiia*	*Acidimicrobiales*	*Acidimicrobiaceae*	*Acidimicrobium*	0.043	0.30±0.13		0.18±0.10
	*Actinobacteria*	*Actinobacteria*	*Actinomycetales*	*Cellulomonadaceae*	*Demequina*	0.029	2.50±1.50		4.03±0.55
	*Actinobacteria*	*Actinobacteria*	*Actinomycetales*	*Nocardioidaceae*	*Kribbella*	0.007	0.94±0.35		0.58±0.14
	*Actinobacteria*	*Actinobacteria*	*Actinomycetales*	*Mycobacteriaceae*	*Mycobacterium*	0.000	1.33±0.59		0.33±0.19
	*Actinobacteria*	*Actinobacteria*	*Actinomycetales*	*Nocardiaceae*	*Rhodococcus*	0.002	0.33±0.13		0.17±0.04
	*Actinobacteria*	*Actinobacteria*	*Actinomycetales*	*Pseudonocardiaceae*	*Saccharopolyspora*	0.000	4.40±1.04		1.57±0.57
	*Actinobacteria*	*Nitriliruptoria*	*Euzebyales*	*Euzebyaceae*	*Euzebya*	0.000	0.51±0.14		0.25±0.04
	*Bacteroidetes*	*Sphingobacteriia*	*Sphingobacteriales*	*Chitinophagaceae*	*Chitinophaga*	0.000	0.27±0.13		0.62±0.21
	*Bacteroidetes*	*Flavobacteriia*	*Flavobacteriales*	*Flavobacteriaceae*	*Flavobacterium*	0.009	1.44±0.35		1.89±0.33
	*Bacteroidetes*	*Flavobacteriia*	*Flavobacteriales*	*Cryomorphaceae*	*Fluviicola*	0.000	0.37±0.22		0.95±0.25
	*Firmicutes*	*Clostridia*	*Clostridiales*	*Clostridiaceae*	*Clostridium*	0.004	0.28±0.05		0.41±0.10
	*Planctomycetes*	*Brocadiae*	*Brocadiales*	*Brocadiaceae*	*Candidatus Scalindua*	0.007	1.01±0.68		1.93±0.55
	*Proteobacteria*	*Alphaproteobacteria*	*Rhodospirillales*	*Acetobacteraceae*	*Acidiphilium*	0.000	1.14±0.51		2.80±0.64
	*Proteobacteria*	*Alphaproteobacteria*	*Sphingomonadales*	*Sphingomonadaceae*	*Kaistobacter*	0.004	0.15±0.30		0.05±0.07
	*Proteobacteria*	*Alphaproteobacteria*	*Sphingomonadales*	*Sphingomonadaceae*	*Novosphingobium*	0.000	0.85±0.29		0.38±0.09
	*Proteobacteria*	*Alphaproteobacteria*	*Rhodobacterales*	*Rhodobacteraceae*	*Rhodobacter*	0.003	1.40±0.25		0.91±0.30
	*Proteobacteria*	*Alphaproteobacteria*	*Sphingomonadales*	*Sphingomonadaceae*	*Sphingobium*	0.002	0.45±0.63		0.10±0.02
	*Proteobacteria*	*Alphaproteobacteria*	*Sphingomonadales*	*Sphingomonadaceae*	*Sphingomonas*	0.000	1.61±0.14		0.56±0.03
	*Proteobacteria*	*Betaproteobacteria*	*Burkholderiales*	*Comamonadaceae*	*Polaromonas*	0.002	0.21±0.15		0.48±0.16
	*Proteobacteria*	*Betaproteobacteria*	*Burkholderiales*	*Oxalobacteraceae*	*Polynucleobacter*	0.043	0.31±0.11		0.44±0.01
	*Proteobacteria*	*Epsilonproteobacteria*	*Campylobacterales*	*Campylobacteraceae*	*Campylobacter*	0.000	3.97±0.81		13.34±5.96
	*Proteobacteria*	*Gammaproteobacteria*	*Legionellales*	*Legionellaceae*	*Legionella*	0.000	0.39±0.10		0.24±0.04
	*Proteobacteria*	*Gammaproteobacteria*	*Xanthomonadales*	*Xanthomonadaceae*	*Luteibacter*	0.002	3.17±0.97		2.00±0.37
	*Synergistetes*	*Synergistia*	*Synergistales*	*Dethiosulfovibrionaceae*	*Dethiosulfovibrio*	0.002	5.20±1.99		2.58±0.88
	*Tenericutes*	*Mollicutes*	*Mycoplasmatales*	*Mycoplasmataceae*	*Mycoplasma*	0.001	0.90±0.18		1.67±0.41
	*Verrucomicrobia*	*Spartobacteria*	*Chthoniobacterales*		*Candidatus Xiphinematobacter*	0.002	1.42±0.27		0.95±0.20
**Soil**	*Proteobacteria*	*Betaproteobacteria*	*Burkholderiales*	*Comamonadaceae*	*Limnobacter*	0.009	0.64±0.17		0.20±0.11
	*Bacteroidetes*	*Sphingobacteriia*	*Sphingobacteriales*	*Flexibacteraceae*	*Pontibacter*	0.016	0.78±0.12		0.45±0.15
**Crop**	*Proteobacteria*	*Gammaproteobacteria*	*Pseudomonadales*	*Pseudomonadaceae*	*Pseudomonas*	0.019	7.76±4.58		2.92±2.24
	*Proteobacteria*	*Gammaproteobacteria*	*Enterobacteriales*	*Enterobacteriaceae*	*Erwinia*	0.004	0.36±0.33		0.09±0.06
	*Proteobacteria*	*Gammaproteobacteria*	*Enterobacteriales*	*Enterobacteriaceae*	*Enterobacter*	0.023	0.37±0.51		0.11±0.10
	*Proteobacteria*	*Gammaproteobacteria*	*Aeromonadales*	*Aeromonadaceae*	*Tolumonas*	0.043	0.30±0.32		0.09±0.07

*Only genera greater and equal to 0.5% differed with a *p*-value < 0.05 (Mann Whitney) are shown.

In soil, at phylum and class levels, no significant differences in the bacterial community were observed between crop areas irrigated with untreated and ClO_2_ treated water, except for the *Betaproteobacteria* class, which showed a reduction of 1.6 folds of their relative abundance. At lower taxonomic levels, the relative abundance of the *Limnobacter* and *Pontibacter* genera significantly decreased in the soil irrigated with ClO_2_ treated water (Table 3). These changes suggest a potentially greater susceptibility to ClO_2_ treatment. Gu et al. [56] reported that the bacterial community in baby spinach shifted significantly after chlorine washing. They observed that *Proteobacteria* species, such as *Stenotrophomonas* spp. and *Erwinia* spp., were relatively tolerant of chlorine treatment, while species of *Flavobacterium* and *Pedobacter* (phylum bacteroidetes) grew rapidly during storage, especially at abusive temperatures. The results obtained suggest that the continuous application of ClO_2_ as a water disinfection treatment to improve the microbiological quality of irrigation water did not cause significant changes in the bacterial community composition of the soil. These results support the use of a low residual concentration of ClO_2_ in the irrigation water, as a corrective measure for water sources of concern, to be applied during the growing cycle without a detrimental impact on the bacterial diversity of soil. Long-term application with subtle but cumulative effects should be considered. Also, previous studies reported that soil irrigated with ClO_2_ treated wastewater did not alter the bacterial community based on a terminal restriction fragment analysis [57]. However, further in-depth studies are still needed to determine the impact of the water disinfection treatments in the bacterial composition across variable water constituent content qualities, duration of treatment and environmental conditions.

For baby spinach samples, no significant differences at the phyla and class levels were found between samples irrigated with ClO_2_ treated and untreated water. However, when the major families and genera (those with average abundance >0.5% in at least one sample) were compared, significant differences were observed between irrigated baby spinach with treated and untreated water. The use of ClO_2_ treated water to irrigate baby spinach during the complete growing cycle significantly reduced the relative abundance of two families and four genera including *Pseudomonas* as well as some less abundant genera such as *Erwinia*, *Enterobacter* and *Tolumonas* (Table 3). In general, these genera were not among the most predominant ones in the bacterial community of baby spinach, except for *Pseudomonas*. This is a notable finding because approximately 30% of the postharvest losses per year in fresh produce have been attributed to the colonization by *Pseudomonas*, *Erwinia* and *Enterobacter* [58–59]. Some species from the *Pseudomonas* and *Erwinia* genera were listed among the top ten plant pathogenic bacteria [60], although *Pseudomonas* and *Erwinia* genera also contain many plant-beneficial groups.

### Impact sources of the bacterial community of baby spinach and their interactions with agronomic habitats

**Fig 3:**
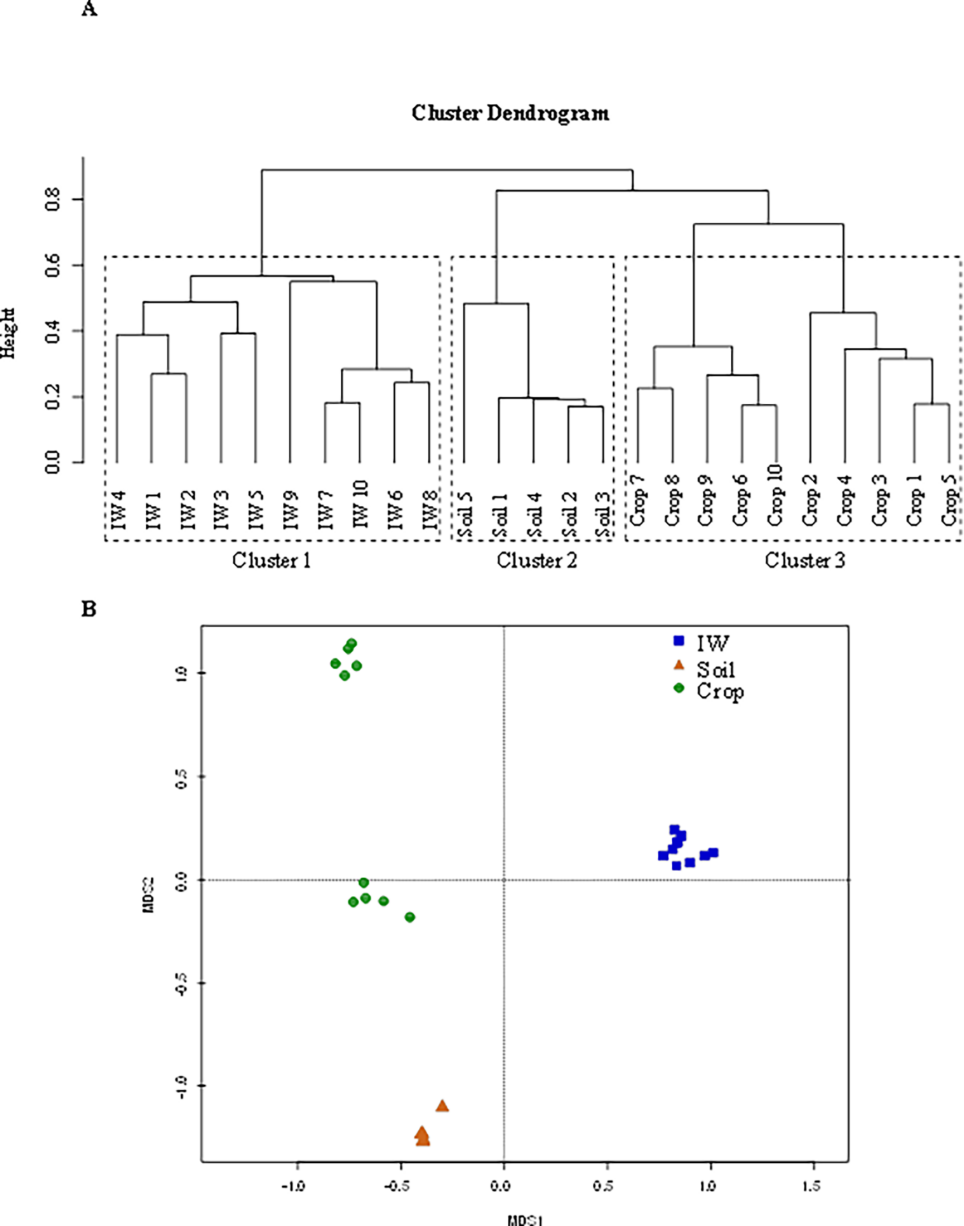
**Dendrogram generated by UPGMA clustering (A) and metric multidimensional scaling (MDS) (B) based on the Bray-curtis distance from the OTUs abundance matrix of irrigation water (IW), soil (Soil) and baby spinach (Crop).** Colors denote the cluster from samples.

Cluster analysis and metric multidimensional scaling (MDS) were performed to evaluate the similarity of the different agronomic habitats (irrigation water, soil and leaves) (Fig 3). The OTU enumeration data were used to construct dissimilarity matrices with Bray-Cutis distance with the aim to determine whether the bacterial community from irrigation water and soil interrelated with the composition of the natural microbiota of baby spinach. The cluster analysis differentiated two clusters; a first one composed by all the irrigation water samples and a second one including two sub-clusters, one for soil samples and another for baby spinach (Fig 3A). The MDS plot displayed similar patterns among samples, in which the bacterial community from baby spinach and soil clustered in close display proximity and differed from those of water samples (Fig 3B). These results suggested that the composition of bacterial community associated with the crop was influenced more by the soil bacterial community than by the irrigation water microbiota. These findings are consistent with previous studies, which suggested that soil can be a source of bacteria associated with leafy greens [25, 61]. In agreement with our results, it was observed that the bacterial community of spinach was more similar to soil microbiota than that of irrigation water [6,62]. This was attributed to the structure of the leaves (open canopy) and very limited vertical separation from the soil seedbed, thereby facilitating dispersal of soil bacteria to the crop. Regarding irrigation water, our results are consistent with previous studies, which demonstrated that the irrigation water bacterial community did not contribute to the variation of the phyllosphere microbial diversity of the crop [11, 10].

This study has a few limitations. The enrolled producer was among the largest baby spinach producers in the study area. This was important to assure that the results of this study are relevant to a large segment of the produced baby spinach in this area of Spain. However, because producers of different sizes may have different management practices caution is needed in extrapolating the results to all producers in the study area. Notably, this study evaluated two growing cycles, where the control and treatment plots were reversed after the first assay to eliminate any factor associated to the specific plots. However, variation in field soil composition may exist thus explaining variations between experiments. However, reversing the control and treatments plots was performed to eliminate any factor associated to the specific plots but this might have caused some effects on the soil bacterial community.

## Conclusions

The results obtained regarding the bacterial community composition and diversity showed that ClO_2_ disinfection treatment positively affected the microbiota of irrigation water reducing–the relative abundance genera associated with spoilage and foodborne illnesses. However, the bacterial community of soil and baby spinach irrigated with ClO_2_ treated water was not demonstrably affected. Small changes were only detected at lower taxonomic levels, particularly for *Pseudomonadaceae* and *Enterobacteriaceae* with a decrease in the abundance of these genera in baby spinach irrigated with ClO_2_ treated water. Based on the results obtained, stabilized ClO_2_ could be considered an eco-compatible disinfection technology as it has a neutral effect on soil and crop microbial diversity.

## Supporting information

S1 Fig Rarefaction curves of sequencing samples. **(A) irrigation water samples (IW), (B) soil samples (soil), (C) baby spinach samples (crop). CT is the control treatments (without chlorine dioxide) and ClO2 represents chlorine dioxide treatments.** (1, IW-CT; 2, IW-CT; 3, IW-CT; 4, IW-CT; 5, IW-CT; 6, IW-CT; 7, IW-CT; 8, IW-CT; 9, IW-CT; 10, IW-CT; 11, IW-ClO_2_; 12, IW-ClO_2_; 13, IW-ClO_2_; 14, IW-ClO_2_; 15, IW-ClO_2_; 16, IW-ClO_2_; 17, IW-ClO_2_; 18, IW-ClO_2_; 19, IW-ClO_2_; 20, IW-ClO_2_. (B) soil samples (1, Soil-CT; 2, Soil-CT; 3, Soil-CT; 4, Soil-CT; 5, Soil-CT; 6, Soil-ClO_2_; 7, Soil-ClO_2_; 8, Soil-ClO_2_; 9, Soil-ClO_2_; 10, Soil-ClO_2_). (C) Baby spinach samples (1, Crop-CT; 2, Crop-CT; 3, Crop-CT; 4, Crop-CT; 5, Crop-CT; 6, Crop-CT; 7, Crop-CT; 8, Crop-CT; 9, Crop-CT; 10, Crop-CT; 11, Crop-ClO_2_; 12, Crop-ClO_2_; 13, Crop-ClO_2_; 14, Crop-ClO_2_; 15, Crop-ClO_2_; 16, Crop-ClO_2_; 17, Crop-ClO_2_; 18, Crop-ClO_2_; 19, Crop-ClO_2_; 20, Crop-ClO_2_).
(TIF)

S1 Table Composition of bacterial families of untreated and ClO_2_ treated samples of irrigation water (IW), soil (Soil) and baby spinach (Crop). Bacterial community is the average of 5 individual samples. Data shown are families that comprised at least 1% of the sequences in at least one sample of a given agronomic habitat.
(PDF)
