# Supplementary figures and images for: Impact of chlorine dioxide disinfection of irrigation water on the epiphytic bacterial community of baby spinach and underlying soil
Source: PLoS One. 2018 Jul 18;13(7):e0199291. doi: 10.1371/journal.pone.0199291 (PMC6051574; doi:10.1371/journal.pone.0199291)

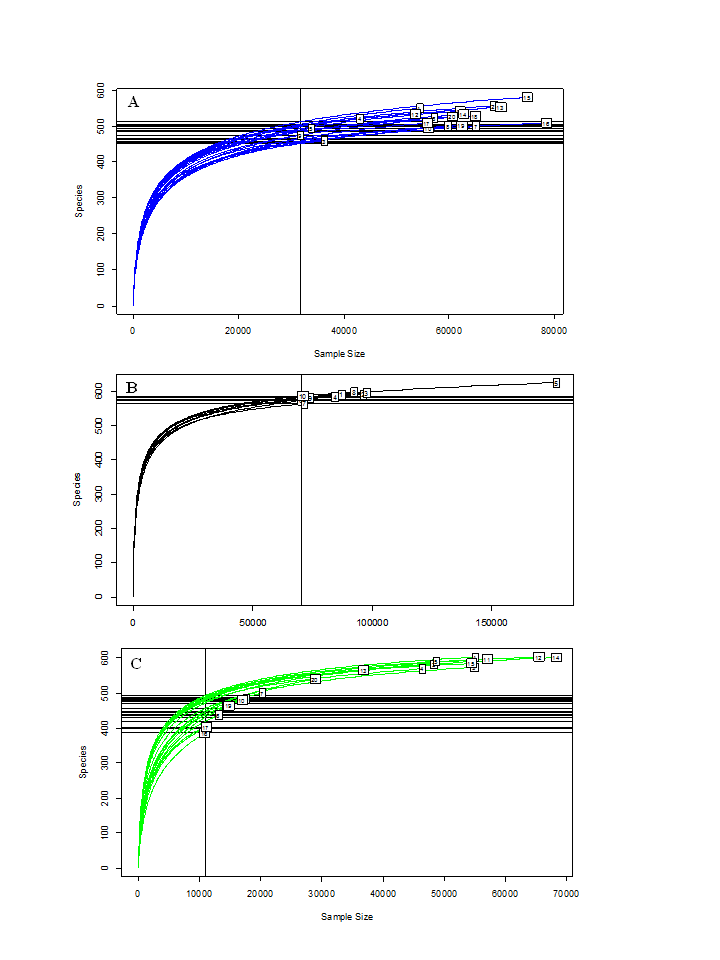

Supplement: S1 Fig — (A) irrigation water samples (IW), (B) soil samples (soil), (C) baby spinach samples (crop). CT is the control treatments (without chlorine dioxide) and ClO2 represents chlorine dioxide treatments. (1, IW-CT; 2, IW-CT; 3, IW-CT; 4, IW-CT; 5, IW-CT; 6, IW-CT; 7, IW-CT; 8, IW-CT; 9, IW-CT; 10, IW-CT; 11, IW-ClO2; 12, IW-ClO2; 13, IW-ClO2; 14, IW-ClO2; 15, IW-ClO2; 16, IW-ClO2; 17, IW-ClO2; 18, IW-ClO2; 19, IW-ClO2; 20, IW-ClO2. (B) soil samples (1, Soil-CT; 2, Soil-CT; 3, Soil-CT; 4, Soil-CT; 5, Soil-CT; 6, Soil-ClO2; 7, Soil-ClO2; 8, Soil-ClO2; 9, Soil-ClO2; 10, Soil-ClO2). (C) Baby spinach samples (1, Crop-CT; 2, Crop-CT; 3, Crop-CT; 4, Crop-CT; 5, Crop-CT; 6, Crop-CT; 7, Crop-CT; 8, Crop-CT; 9, Crop-CT; 10, Crop-CT; 11, Crop-ClO2; 12, Crop-ClO2; 13, Crop-ClO2; 14, Crop-ClO2; 15, Crop-ClO2; 16, Crop-ClO2; 17, Crop-ClO2; 18, Crop-ClO2; 19, Crop-ClO2; 20, Crop-ClO2). (TIF) [file pone.0199291.s001.tif]
