# Supplementary material for: Impact of chlorine dioxide disinfection of irrigation water on the epiphytic bacterial community of baby spinach and underlying soil
Source: PLoS One. 2018 Jul 18;13(7):e0199291. doi: 10.1371/journal.pone.0199291 (PMC6051574; doi:10.1371/journal.pone.0199291)
Supplement: S1 Table — Bacterial community is the average of 5 individual samples. Data shown are families that comprised at least 1% of the sequences in at least one sample of a given agronomic habitat. (PDF) [file pone.0199291.s002.pdf]

	IW	IW-ClO_2	Soil	Soil-ClO_2	Crop	Crop-ClO_2
Acetobacteraceae	1.46±0.50	3.18±0.64				
Aeromonadaceae					0.30±0.01	0.10±0.07
Alcaligenaceae	1.37±0.35	1.33±0.67				
Alteromonadaceae					0.82±0.20	0.35±0.03
Bacillaceae	0.89±0.34	2.69±0.76	13.49±1.45	14.29±1.37	12.79±3.76	14.86±3.10
Bifidobacteriaceae	2.76±1.10	3.03±0.88				
Brocadiaceae	1.01±0.64	1.93±0.53				
Campylobacteraceae	4.07±0.66	13.50±5.69				
Caulobacteraceae	1.45±0.25	1.12±0.33				
Caulobacteraceae					0.81±0.59	0.80±0.55
Cellulomonadaceae	2.59±1.42	4.04±0.52	0.83±0.05	0.87±0.13	0.75±0.30	0.80±0.39
Chitinophagaceae	0.90±0.18	1.34±0.29				
Chromatiaceae	0.95±0.55	0.70±0.57			0.87±0.09	0.57±0.04
Chthoniobacterales	1.42±0.25	0.95±0.20				
Clostridiaceae					0.91±0.47	1.25±0.11
Comamonadaceae	4.61±1.78	5.97±1.43	1.31±0.17	0.52±0.11	2.53±0.60	1.86±0.76
Conexibacteraceae			1.45±0.16	1.33±0.22		
Cryomorphaceae	0.37±0.21	0.95±0.24				
Desulfobacteraceae	0.90±0.27	0.73±0.24				
Desulfovibrionaceae			1.06±0.13	1.04±0.14		
Desulfuromonadaceae					0.87±0.79	0.89±0.75
Dethiosulfovibrionaceae	5.20±1.88	2.58±0.84				
Enterobacteriaceae					0.95±0.08	0.37±0.05
Enterococcaceae	1.04±0.56	0.69±0.31				
Erythrobacteraceae					0.38±0.27	0.37±0.36
Flavobacteriaceae	2.12±0.58	2.58±0.42	0.73±0.15	1.04±0.61	1.36±0.11	0.77±0.57
Flexibacteraceae			1.44±0.19	1.00±0.20	0.91±0.46	0.71±0.24
Geodermatophilaceae					0.90±0.17	0.97±0.34
Heliobacteriaceae					4.05±1.84	4.32±1.90
Hyphomicrobiaceae			0.79±0.09	0.97±0.17	0.69±0.46	0.76±0.47
Methylophilaceae					0.69±0.06	0.49±0.05
Microbacteriaceae			0.91±0.11	0.94±0.16	0.83±0.38	0.76±0.45
Micrococcaceae			9.72±1.35	9.81±2.59	3.23±1.16	3.18±1.58
Micromonosporaceae			1.14±0.23	1.28±0.26		
Moraxellaceae	1.72±0.20	0.62±0.24			1.15±0.79	2.59±0.58

Mycobacteriaceae	1.33±0.56	0.33±0.18				
Mycoplasmataceae	0.90±0.17	1.67±0.40				
Nocardioidaceae	1.04±0.34	0.69±0.14	10.38±0.61	10.02±0.74	3.85±0.81	3.61±0.25
Oxalobacteraceae	1.03±0.51	1.34±0.61			0.81±0.62	0.60±0.29
Paenibacillaceae	0.39±0.05	1.32±0.06	2.54±0.20	2.73±0.25	2.20±0.64	2.47±0.65
Phyllobacteriaceae					0.72±0.57	0.81±0.69
Piscirickettsiaceae					0.34±0.32	0.74±0.83
Planococcaceae					2.17±1.80	4.14±1.87
Pseudomonadaceae	1.20±0.33	0.39±0.19			7.96±4.59	3.47±3.60
Pseudonocardiaceae	4.83±1.06	2.17±0.57	3.68±0.31	4.06±0.22	1.60±0.52	1.82±0.80
Rhizobiaceae	0.77±0.07	0.94±0.09			0.84±0.13	0.65±0.50
Rhodobacteraceae	2.22±0.57	1.46±0.52	1.34±0.16	1.36±0.31	1.78±0.68	2.13±0.62
Rhodospirillaceae	0.94±0.05	1.00±0.48	3.99±0.54	3.62±0.62	1.96±0.83	2.02±1.15
Rickettsiaceae	1.62±0.55	2.07±0.98			2.51±0.07	3.08±0.05
Saprospiraceae	4.70±1.13	3.78±0.78				
Sphingobacteriaceae	0.81±0.08	0.93±0.17			0.89±0.18	0.23±0.02
Sphingomonadaceae	3.17±0.21	1.12±0.27	3.15±0.16	3.13±0.35	4.51±1.12	4.83±1.75
Staphylococcaceae	0.13±0.02	0.51±0.06				
Streptomycetaceae			7.21±1.44	7.30±1.43	1.52±0.66	1.62±0.65
Streptosporangiaceae			0.75±0.15	0.67±0.11		
Thermoactinomycetaceae			1.21±0.11	1.50±0.22	1.17±0.47	1.19±0.37
Thermoanaerobacteraceae			1.17±0.11	1.29±0.09		
Thermogemmatisporaceae	8.78±3.82	7.85±5.22			3.02±0.67	3.17±0.74
Thermomonosporaceae			0.88±0.11	0.91±0.09		
Verrucomicrobiaceae	4.15±0.82	2.56±0.41				
Xanthomonadaceae			1.84±0.12	2.05±0.38	2.16±0.78	2.07±0.91
